# Structural Basis for the Selective Inhibition of Cdc2-Like Kinases by CX-4945

**DOI:** 10.1155/2019/6125068

**Published:** 2019-08-18

**Authors:** Joo Youn Lee, Ji-Sook Yun, Woo-Keun Kim, Hang-Suk Chun, Hyeonseok Jin, Sungchan Cho, Jeong Ho Chang

**Affiliations:** ^1^Department of Biology Education, Kyungpook National University, 80 Daehak-ro, Buk-gu, Daegu 41566, Republic of Korea; ^2^Biosystem Research Group, Korea Institute of Toxicology, 141 Gajeong-ro, Yuseong-gu, Daejeon 34114, Republic of Korea; ^3^Research Institute for Phylogenomics and Evolution, Kyungpook National University, 80 Daehak-ro, Buk-gu, Daegu 41566, Republic of Korea; ^4^Natural Medicine Research Center, Korea Research Institute of Bioscience and Biotechnology, 30 Yeongudanji-ro, Ochang-eup, Cheongju-si, Chungbuk 28116, Republic of Korea; ^5^Department of Biomolecular Science, Korea University of Science and Technology, 217 Gajeong-ro, Gajeong-dong, Yuseong-gu, Daejeon 34113, Republic of Korea

## Abstract

Cdc2-like kinases (CLKs) play a crucial role in the alternative splicing of eukaryotic pre-mRNAs through the phosphorylation of serine/arginine-rich proteins (SR proteins). Dysregulation of this processes is linked with various diseases including cancers, neurodegenerative diseases, and many genetic diseases. Thus, CLKs have been regarded to have a potential as a therapeutic target and significant efforts have been exerted to discover an effective inhibitor. In particular, the small molecule CX-4945, originally identified as an inhibitor of casein kinase 2 (CK2), was further revealed to have a strong CLK-inhibitory activity. Four isoforms of CLKs (CLK1, CLK2, CLK3, and CLK4) can be inhibited by CX-4945, with the highest inhibitory effect on CLK2. This study aimed to elucidate the structural basis of the selective inhibitory effect of CX-4945 on different isoforms of CLKs. We determined the crystal structures of CLK1, CLK2, and CLK3 in complex with CX-4945 at resolutions of 2.4 Å, 2.8 Å, and 2.6 Å, respectively. Comparative analysis revealed that CX-4945 was bound in the same active site pocket of the CLKs with similar interacting networks. Intriguingly, the active sites of CLK/CX-4945 complex structures had different sizes and electrostatic surface charge distributions. The active site of CLK1 was somewhat narrow and contained a negatively charged patch. CLK3 had a protruded Lys248 residue in the entrance of the active site pocket. In addition, Ala319, equivalent to Val324 (CLK1) and Val326 (CLK2), contributed to the weak hydrophobic interactions with the benzonaphthyridine ring of CX-4945. In contrast, the charge distribution pattern of CLK2 was the weakest, favoring its interactions with benzonaphthyridine ring. Thus, the relatively strong binding affinities of CX-4945 with CLK2 are consistent with its strong inhibitory effect defined in the previous study. These results may provide insights into structure-based drug discovery processes.

## 1. Introduction

The serine/arginine-rich protein (SR protein) is a well-conserved trans-acting protein that plays an important role in the splicing regulation of eukaryotic genes [1]. SR proteins are particularly crucial for the selection of splice site through the elaborate and complex interplay with other splicing factors and* cis*-acting elements on the pre-mRNAs. SR proteins are generally composed of one or two RNA recognition motifs (RRMs) at the N-terminus, and two arginine/serine-rich domains (RS1 and RS2 domains) at the C-terminus [2]. The RRM of the SR protein is involved in RNA binding, and the RS domains function by recruiting several proteins involved in splicing [3]. Especially, the phosphorylation of SR protein at its RS domain is crucial for the splicing regulation through the alteration in the protein-protein and protein-RNA interactions as well as in the subcellular localization. As the SR protein is phosphorylated, the splicing-promoting and -inhibitory factors are recruited to the phosphorylated RS domains through the protein-protein interaction and control the selective splicing of the pre-mRNA [4]. On the other hand, heterogeneous nuclear ribonucleoproteins (hNRNPs) interact with splicing-inhibitory factors through RNA-dependent motifs, which lack the RS domain, and compete with SR proteins [5]. SR proteins are phosphorylated mainly by cdc2-like kinase (CLK) and serine/arginine-rich protein kinase (SRPK) [6]. They are phosphorylated at the serines on the N-terminal region of the RS domain mostly by SRPK1 in the cytoplasm and then transported into the nucleus by a carrier protein such as transportin-SR2 [7]. Therein, the remaining serines are further phosphorylated by CLK. In the SR protein regulation, SRPK1 and CLK1 seem to partition their activities for SR protein phosphorylation based on Ser-Pro versus Arg-Ser placement rather than on N- and C-terminal preferences along the RS domain [8, 9].

CLKs are dual-specificity kinases that are activated by autophosphorylating their own tyrosine residues and subsequently phosphorylate the serine/threonine residues of the SR protein [10]. CLKs are conserved in eukaryotes with different annotations including KNS1 in* Saccharomyces cerevisiae*, AFC1-3 in* Arabidopsis thaliana*, DOA in* Drosophila melanogaster*, and CLK/STY in* Mus musculus*, and four isoforms of CLKs (CLK1, CLK2, CLK3, and CLK4) have been reported for* Homo sapiens* [11]. DOA is involved in various stages of development including differentiation and maintenance, and human CLKs regulate alternative splicing [11]. CLK1 is present in various neurons and is involved in Alzheimer's disease-linked neuronal differentiation, and CLK3 is found in germ cells involved in spermatogenesis [12].

The hypophosphorylation or hyperphosphorylation of SR protein can induce abnormal splicing, possibly causing several diseases [13, 14]. Thus, the regulation of SR protein phosphorylation could contribute to the treatment of various splicing-related diseases as well as inhibit the growth of cells such as cancer cells [15]. Even though a few CLK inhibitors (including TG-003 and KH-CB19) and SRPK inhibitors (including SRPIN340) are available, their clinical potential for splicing-related diseases needs to be carefully evaluated [16, 17]. Therefore, the development of more selective and potent inhibitors of CLK or SRPK is required.

CK2 is a serine/threonine kinase required for signal transduction in endothelial cell proliferation, metastasis, and apoptosis associated with infectious diseases and cancers [18, 19]. Since CK2 is overexpressed in various human carcinomas and its strong correlation with tumorigenesis has been increasingly reported in the cellular and animal systems, CK2 was regarded as a promising anti-cancer target and significant efforts has been exerted to discover an effective inhibitor of CK2 Among many different CK2 inhibitors developed so far, CX-4945 is one of the most potent and selective one that exerts antiproliferative and antiangiogenic activities in cancer cells, and anti-tumor activity in xenograft mouse model [20]. Its strong antiproliferative activity could be explained by the inhibition of cell cycle and PI3K/AKT signaling. In addition, CX-4945 suppressed the angiogenesis through the inhibition of HIF1*α* transcription Currently, CX-4945 is underway of clinical trial, in combination with gemcitabine and cisplatin for the frontline treatment of individuals with bile duct cancers (cholangiocarcinoma).

Later, CX-4945 was revealed to have a strong inhibitory activity on CLKs; IC_50_ values of 82.3 nM (CLK1), 3.8 nM (CLK2), and 90.0 nM (CLK3). [21] This could be explained by the phylogenetic similarity of CLKs with CK2. Both of them are classified as the CMGC kinase superfamily. Out of CLKs, CLK2 was most strongly inhibited by CX-4945 with 3.8 nM of IC_50_* in vitro*, which is quite noteworthy in that most of CLK inhibitors are more potent on CLK1/CLK4 than CLK2. Moreover, its inhibitory potency on CLK2 was similar to or even higher than that on CK2 (14.7 nM of IC_50_). Treatment with CX-4945 caused a significant suppression of SR protein phosphorylation in mammalian cells, leading to a widespread alteration in alternative splicing of numerous genes

In order to elucidate the selective inhibitory effect of CX-4945 on CLKs, we determined the structures of CLK1, CLK2, and CLK3 in complex with CX-4945. In addition, we compared the complex structures with the structure of CK2. Our findings could provide insights into the structural basis of effective drugs designed for the treatment of many diseases associated with splicing defects.

## 2. Materials and Methods

### 2.1. Construction of CLKs

The CLK1, CLK2, and CLK3 genes were obtained from a cDNA library of the human 293T cell line by PCR with pfu-X (SPX95-E500; Solgent, Republic of Korea). To prepare N-terminally truncated constructs, the amplified CLK genes with each set of primers were digested with restriction enzymes: CLK1 (residues 148–484) with NheI (R016S; Enzynomics, Republic of Korea) and XhoI (R0075; Enzynomics, Republic of Korea), CLK2 (residues 125–488) with NheI and HindIII (Enzynomics, Republic of Korea), CLK3 (residues 127–484) with NdeI and HindIII (R0065; Enzynomics, Republic of Korea). The digested genes were ligated to both the pET28a and pET24d vectors with the T4 ligase (M0202S; Roche, Germany) at 18°C for 16 h. The ligated plasmids were transformed into the* Escherichia coli* DH5*α* strain, and the transformants were confirmed by colony PCR. The recombinant genes were verified by DNA sequencing.

### 2.2. Expression and Purification of Recombinant CLK Proteins

The CLK-encoding plasmids were transformed into the* E. coli* strain BL21 (DE3). The cells were cultured at 37°C using 9 L of Luria-Bertani medium (L4488; MB Cell, Republic of Korea) containing 50 mg/L kanamycin (A1493; AppliChem, Germany) until an OD_600_ of approximately 0.7. Following induction with 0.3 mM isopropyl *β*-D-1-thiogalacto-pyranoside (420322; Calbiochem, Germany), the cells were further grown at 20°C for 16 h. The cultured cells were harvested by centrifugation at 5,000* g* for 20 min at 4°C, and the pellet was resuspended in 20 mM Tris pH 8.0 (T1895; Sigma-Aldrich, USA), 250 mM NaCl (A2942; AppliChem, USA), 5% glycerol (56515; Affymetrix, USA), 0.2% Triton-X 100 (9002931; Sigma-Aldrich, USA), 0.2 mM phenylmethylsulfonyl fluoride (P7326; Sigma-Aldrich, USA), and 10 mM *β*-mercaptoethanol (60242; Bio Basic, Canada). The cells were disrupted by ultrasonication, and the cell debris was removed by centrifugation at 11,000* g* for 50 min. The lysate was bound to Ni-NTA agarose resin (30230; Qiagen, Germany) for 90 min at 4°C. After washing with buffer (A) containing 50 mM Tris pH 8.0, 500 mM NaCl, and 20 mM imidazole (I5513; Sigma-Aldrich, USA), the bound proteins were eluted with 250 mM imidazole in buffer (A). Size-exclusion chromatography (SEC) was performed on the purified CLK1 protein using HiPrep 16/60 Sephacryl S-300 HR (17116701; GE Healthcare, UK) with a buffer containing 20 mM Tris pH 7.5, 150 mM NaCl, and 2 mM dithiothreitol (233155; Calbiochem, Germany). For the CLK2 and CLK3 proteins, the SEC buffer also contained 2 mM L-arginine (74793; DAEJUNG, Republic of Korea) and L-glutamic acid (G1501; Sigma-Aldrich, USA) as used in a previous report [23]. The purified proteins were confirmed by SDS-PAGE and concentrated using Amicon Ultra 30K Centrifugal Filters (Merck Millipore, USA) to 20 mg/mL. The resulting protein was stored at −80°C in an aliquot.

### 2.3. Crystallization

All crystallization trials were performed at 20°C using the sitting-drop vapor diffusion method in 96-well sitting-drop crystallization plates (102000100; Art Robbins Instruments, USA). Over 480 different conditions from sparse-matrix screening solution kits were used to identify crystallization conditions. The kits used included PEG/Ion (HR2-126 and -098), Index (HR2-144), Crystal Screen 1/2 (HR2-110 and -112), and SaltRx 1/2 (HR2-107 and -109) from Hampton Research (USA) and Wizard (CS-311) from Jena Bioscience (Germany). All CLK crystals were improved or optimized using an additive screening kit (HR2-428; Hampton Research, USA) and detergent screening kit (HR2-406; Hampton Research, USA). CLK1 crystals were grown for 7 days with 8% Tacsimate pH 8.0 (HR2-829; Hampton Research, USA), 18% w/v polyethylene glycol 3350 (1546547; Sigma-Aldrich, USA), and 3% v/v glycerol. CLK2 crystals were grown for 4 days with 0.1 M Bis-Tris pH 5.5 (B9754; Sigma-Aldrich, USA), 0.2 M MgCl_2_ (M8266; Sigma-Aldrich, USA), and 25% w/v polyethylene glycol 3350 (1546547; Sigma-Aldrich, USA). The crystals of CLK3 were grown for 2 days with 60% Tacsimate pH 7.0 (HR2-755; Hampton Research, USA), 0.025% dichloromethane (CH_2_Cl_2_; 270997, Sigma-Aldrich, USA). The CX-4945 complexes were obtained by soaking CLK crystals with 10–20 mM CX-4945 (Selleckchem) using dimethyl sulfoxide (D4540; Sigma-Aldrich, USA) for 1–6 h. Crystals were stabilized using cryoprotectants containing 30% glycerol and then flash frozen in liquid nitrogen for diffraction analysis.

### 2.4. Diffraction Data Collection and Structure Determination

The diffraction data of the CLK1/CX-4945, CLK2/CX-4945, and CLK3/CX-4945 complexes were collected using a Quantum 270 CCD detector (ADSC, San Diego, CA, USA) at 7A beamline at the Pohang Accelerator Laboratory (Republic of Korea). The collected diffraction data were processed using the HKL-2000 suite program [24]. Crystals of the CLK1/CX-4945 complex belonged to space group* P*2_1_ and diffracted to 2.7 Å resolution. In contrast, the CLK2/CX-4945 complex belonged to space group* P*4_3_2_1_2 and diffracted to 2.8 Å resolution. The CLK3/CX-4945 complex also belonged to space group* I*222 and diffracted to 2.6 Å resolution. The crystal structures were determined by molecular replacement methods using PHENIX software version 1.9-1692 (PHENIX) [25]. The structure of CLK1 from the CLK1/10Z-hymenialdisine complex (PDB code: 1Z57) was used as a search model to obtain phase information for the CLK1/CX-4945 complex [23]. The CLK1/CX-4945 structure was used as a starting model to determine the structures of the CLK2/CX-4945 and CLK3/CX-4945 complexes. Model construction of all three structures was performed using the Coot program [26], and model structures were refined using PHENIX. The results from data collection and statistical analyses are shown in Table 1. All structures were produced with PyMOL (www.pymol.org).

## 3. Results and Discussion

### 3.1. Overall Structures of CLK/CX-4945 Complexes

**Figure 1:**
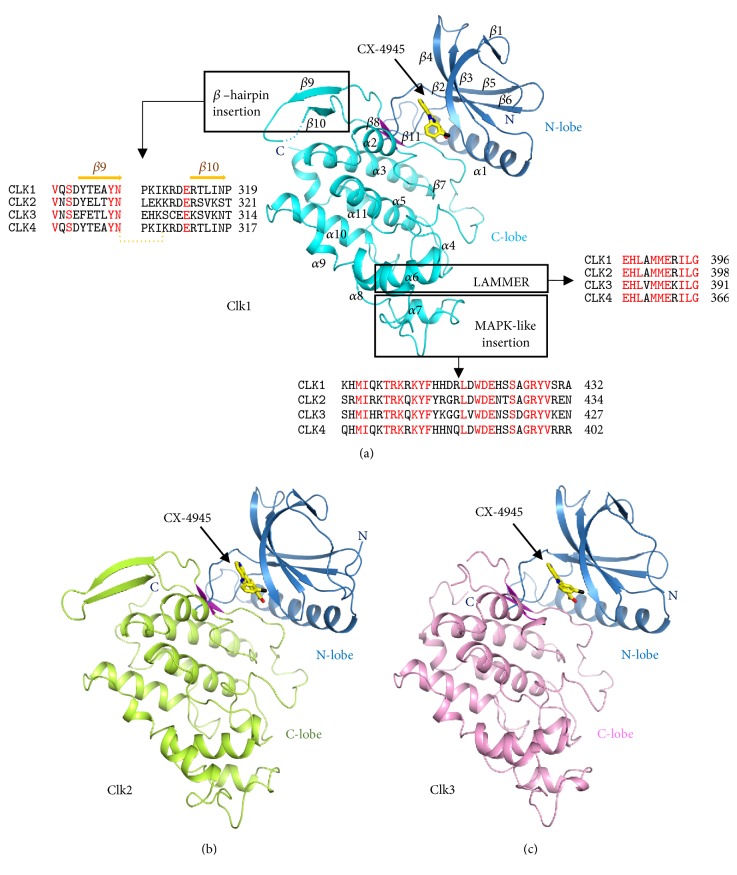
*Overall structures of CLK/CX-4945 complexes*. (a) Overall structure of the CLK1/CX-4945 complex. Both the N-lobe and C-lobe are colored in cyan and blue, respectively. The ligand CX-4945 is shown in yellow with nitrogen, oxygen, and chlorine colored in blue, red, and black, respectively. The common features of CLK (EHLAMMERILG motif, MAPK-like insertion, and *β*-hairpin insertion) are indicated by black boxes, and the amino acid sequences corresponding to these parts are shown below. (b) Overall structure of the CLK2/CX-4945 complex. The N-lobe and C-lobe are colored in blue and light green, respectively. CX-4945 is shown in yellow. (c) Overall structure of the CLK3/CX-4945 complex. The N-lobe and C-lobe are colored in blue and pink, respectively. The structures of CLK1, CLK2, and CLK3 are presented in the same orientation.

The three isoforms of human CLK proteins showed high sequence homology (Supp_Figure 1). The sequence identities for human CLK1-CLK2, CLK1-CLK3, and CLK2-CLK3 were 54%, 49%, and 60%, respectively. Extensive structural analysis performed in this study revealed that CLK1, CLK2, and CLK3 shared structural similarities as well. The overall structure of the CLK catalytic domain contained 12 *β*-strands and 10 *α*-helices that could be divided into the N-lobe and C-lobe, which are typical forms of the kinase (Figure 1(a)). In particular, most *β*-strands and *α*-helices were localized in the N-terminal and C-terminal regions, respectively. The N-lobe consisted of an *α*1 helix followed by four *β*-strands (*β*1, *β*2, *β*3, *β*4) and another two *β*-strands (*β*4, *β*5). The C-lobe had three conserved insertions, which were the EHLAMMERILG motif (also called LAMMER kinase), mitogen-activated protein kinase (MAPK)-like insertion, and *β*-hairpin insertion. The EHLAMMERILG motif was found at residues 386–396 of CLK1, 388–398 of CLK2, and 381–391 of CLK3. The MAPK-like insertion was found at residues 400–432 of CLK1, 402–434 of CLK2, and 395–427 of CLK3. The *β*-hairpin insertion should be located at residues 300–319 of CLK1, residues 302–321 of CLK2, and 295–314 residues of CLK3; however, these parts were partially disordered in CLK1 and fully disordered in CLK3 (Figures 1(a)–1(c)).

**Figure 2:**
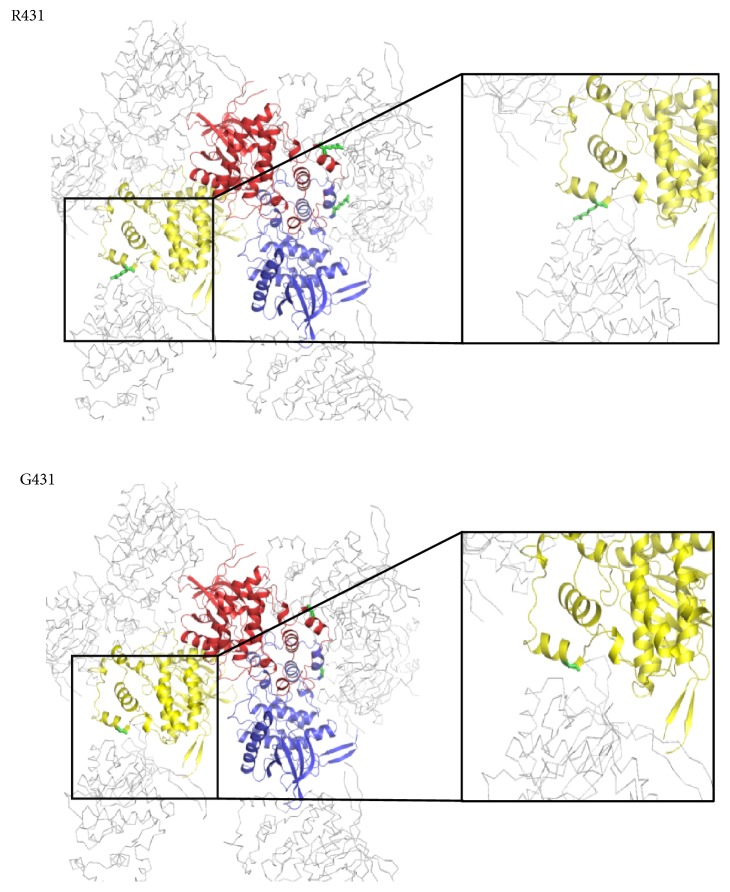
*Comparison of crystal packing between the wild-type (R431) and mutant (G431) molecules of CLK1*. The trimeric CLK1 molecules in the asymmetric unit are colored in red, yellow, and blue. The neighbored symmetry-related molecules are presented as gray C*α* ribbons. The side chains of Arg431 and Gly431 are shown in a green stick model. The detailed view of the packing interface is amplified in a black box.

In this study, for CLK1/CX-4945 complex, we used the CLK1 protein containing a single amino acid mutation (R431G) by chance. Thus, the wild-type CLK1 protein was not crystallized despite our intensive trial with various experimental conditions. Based on the results, we examined the effect of the mutation on the crystallographic packing of the CLK1/CX-4945 complex. We speculate that the side chain of Arg431 might negatively affect molecular packing during crystal formation due to its flexibility. Although there are polar residues (Tyr199, Ser328, and the main chain of Ala198) in the vicinity of the Arg431 residue, no interaction could be formed due to the large distance of 5.8 Å (Figure 2). Moreover, the mutation point was far from the active site; thus, we speculated that R431G mutation was unlikely to affect the active site conformation and binding ability of CX-4945.

### 3.2. Comparison of CLK/CX-4945 Structures

**Figure 3:**
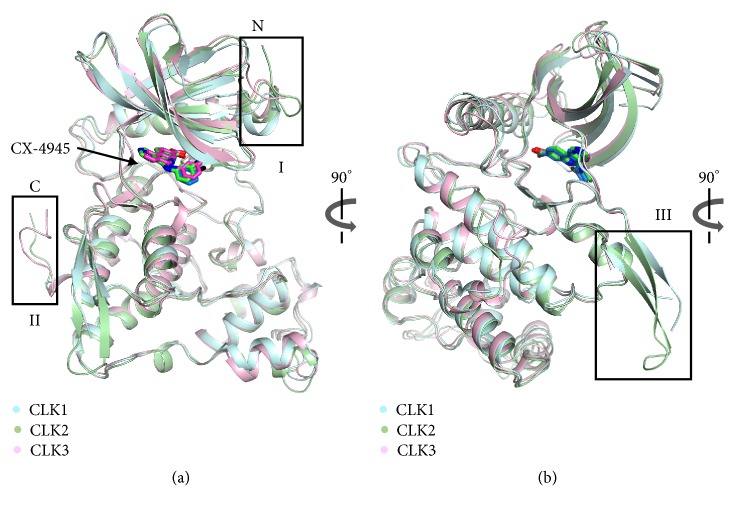
*Structural comparison of CLKs*. (a) The superimposed structures of CLK1/CX-4945, CLK2/CX-4945, and CLK3/CX-4945 indicated three discrete parts; the N- and C-termini are indicated as I and II (black boxes). The CLK1 and bound CX-4945 molecules are colored in cyan and blue, respectively. The CLK2 and bound CX-4945 molecules are colored in light green and green, respectively. The CLK3 protein and CX-4945 ligand are colored in pink and magenta, respectively. (b) The 90° rotated view along the Y-axis from (a) is shown with the varied *β*-hairpin insertion conformations indicated as III (black box).

Structural differences among the CLK/CX-4945 complexes were evaluated using a web-based Dali server [27]. The Z-score and root-mean-square deviation (r.m.s.d.) values indicated the similar conformation of all three structures; the values for CLK1/CX-4945 and CLK2/CX-4945 were 44.8 and 1.5 Å (322 C*α* positions aligned), the values for CLK1/CX-4945 and CLK3/CX-4945 were 43.6 and 1.5 Å (318 C*α* positions aligned), and the values for CLK2/CX-4945 and CLK3/CX-4945 were 46.9 and 1.1 Å (324 C*α* positions aligned). Superposition of the three complexes revealed three discrete conformations in the N- and C-termini and *β*-hairpin insertion structure (Figure 3). CLK2 had the longest N-terminus with an extra eight residues; in contrast, CLK1 had the shortest N-terminus (Figure 3(a)). CLK3 had the longest C-terminus with an extra seven residues compared with the C-terminus of CLK1. For the *β*-hairpin insertion structure, CLK2 had a complete conformation, whereas CLK1 showed a partially disordered loop (Figure 3(b)). In contrast, CLK3 did not have the entire *β*-hairpin insertion structure due to its flexibility. We also examined structural differences using the ligand KH-CB19, another CLK inhibitor previously reported [28]. The CLK1/CX-4945 and CLK1/KH-CB19 complexes were aligned with a r.m.s.d. of 0.9 Å for 327 C*α* positions, and the CLK3/CX-4945 and CLK3/KH-CB19 complexes were aligned with a r.m.s.d. of 1.1 Å for 326 C*α* positions (Supp_Figure 2).

### 3.3. Comparison of the CX-4945 Binding Site

**Figure 4:**
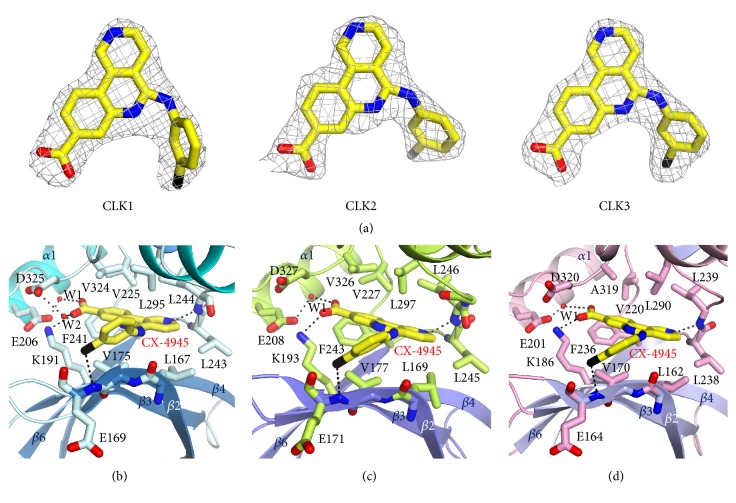
*CX-4945 binding sites of CLKs*. (a) The 2Fo-Fc electron densities of the CX-4945 molecules bound in CLK1, CLK2, and CLK3 are shown at the 1.0 *σ* contoured level. (b) Active site residues in coordination with CX-4945 (yellow) in the structure of the CLK1/CX-4945 complex. The small red spheres (W1 and W2) indicate water molecules. Dashed black lines represent interactions between ligands and residues. Nitrogen, oxygen, and chlorine are colored in blue, red, and black, respectively. The N-lobe and C-lobe are colored in cyan and blue, respectively. All residues are shown in light cyan. (c) Binding pocket of CLK2/CX-4945 in coordination with a CX-4945 molecule (yellow). The small red sphere (W1) indicates a water molecule. The N-lobe and C-lobe are colored in light green and purple, respectively. All residues are shown in light green. (d) CLK3/CX-4945 in coordination with a CX-4945 molecule (yellow) and a water molecule (W1) in the active site. The N-lobe and C-lobe are colored in pink and light purple, respectively. All residues are shown in pink.

To determine the binding mode of CX-4945, we compared the conformations of the CX-4945-bound active sites of CLKs. The electron density of CX-4945 is clearly shown in each of the CLK structure (Figure 4(a)). In the same orientation, the conformations of 3-chlorophenyl rings were varied, whereas the benzonaphthyridine rings were well superimposed. This might be attributed to the flexibility of connecting loops between the N-lobe and C-lobe that resulted in the slightly different locations of *β*2 strands. Therefore, the altered positions of the main chain of Glu171, which interacted with the chlorine of the 3-chlorophenyl ring, induced conformational changes in the CX-4945 ligands (Figures 4(b)–4(d)).

Detailed views of the active sites of the CLK/CX-4945 structures indicated the CX-4945 molecule was tightly bound in the pocket of CLKs mainly by hydrophobic interactions between the benzonaphthyridine ring and nonpolar residues from both the N-lobe and C-lobe. In the CLK1/CX-4945 complex, the carboxy group of CX-4945 directly interacted with Lys191 located in the *β*4 strand and formed water-mediated interactions with Glu206 and Asp325 in the *α*1 helix by hydrogen bonding (Figure 4(b)). The 2N of naphthyridine ring interacted with the main chain of Leu244 by hydrogen bonding. In addition, as mentioned previously, the chlorine of the phenyl ring interacted with the main chain of Glu169 in the *β*2 strand. Val225, Leu244, Leu295, and Val324 of the C-lobe and Val167, Val175, and Phe241 of the N-lobe interacted with benzonaphthyridine by hydrophobic stacking interactions. The interactions between CX-4945 and CLK2 were highly similar except for the carboxyl group of CX-4945. The carboxyl group also directly interacted with Lys193, and water-mediated interactions were observed only with Glu208 and not Asp327 because there was no water molecule between them (Figure 4(c)). The active site environment of CLK3/CX-4945 was also similar to that of CLK2/CX-4945 except for the valine residue (corresponding to Val324 and Val326 in CLK1 and CLK2, respectively), which was replaced with the Ala319 residue (Figure 4(d)). This substitution may contribute to weaker hydrophobic interactions (see below). The results are consistent with the findings of previous studies showing that CX-4945 more strongly inhibits CLK2 than CLK3 [21].

### 3.4. Selective Inhibitory Potency of CX-4945 against CLKs

CX-4945 had stronger inhibitory potency toward CLK2 and weaker inhibitory effect toward CLK3; thus, structural analysis was required to investigate the different inhibitory effects despite the highly similar structures of CLKs [21, 29].

**Figure 5:**
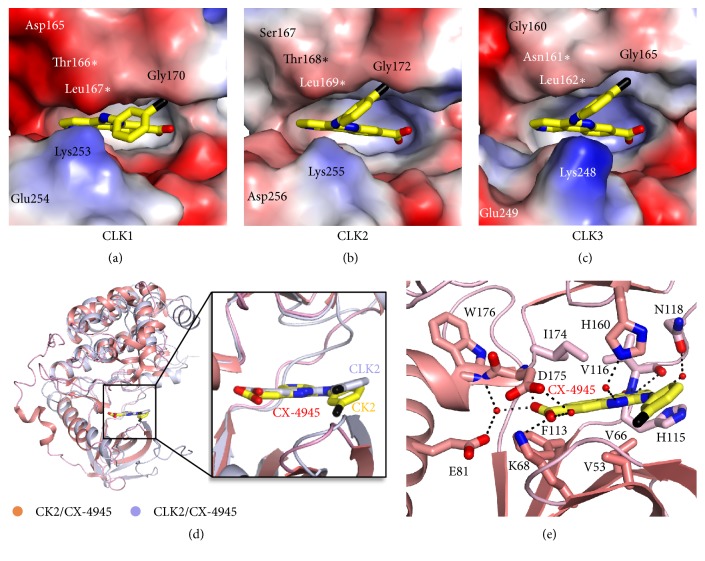
*Comparison of the CX-4945 binding mode*. (a) Electrostatic surface representation of the CX-4945 binding pocket of CLK1. The bound CX-4945 is shown in yellow. The residues surrounding the binding pocket are labeled. The asterisk indicates the main chain of the corresponded residue. The blue and red colors represent positive and negative charges, respectively. (b) Electrostatic surface representation of the CX-4945 binding pocket of CLK2. The bound CX-4945 is shown in yellow. The residues surrounding the binding pocket are labeled. The asterisk indicates the main chain of the corresponded residue. (c) Electrostatic surface representation of the CX-4945 binding pocket of CLK3. The bound CX-4945 is shown in yellow. The residues surrounding the binding pocket are labeled. The asterisk indicates the main chain of the corresponded residue. (d) Superposition of the CLK2/CX-4945 and CK2/CX-4945 (PDB code; 3PEI) complexes. The CLK2/CX-4945 complex is colored in light purple. In the CK2/CX-4945 complex, *α*-helices and *β*-sheets are colored in salmon, and loops are colored in pink. The detailed view of bound CX-4945 is shown in a square box (yellow for CK2 and light purple for CLK2). The positions of the bound CX-4945 in CLK2 and CK2 are almost identical. (e) The CK2/CX-4945 complex in coordination with a CX-4945 molecule (yellow) and water molecules in the active site. The residues of both *α*-helices and *β*-sheets are shown in salmon, and one loop is colored in pink. The four small red spheres represent water molecules. Hydrogen bonds between CK2 and CX-4945 are shown as black dashed lines.

Accordingly, we analyzed the active sites of CLKs. The electrostatic surface representation of the active site indicated that CX-4945 molecules were well fitted in the active site pocket (Figure 5). However, the shape and charge distribution were somewhat different depending on the CLKs. CLK1 had the smallest and CLK3 had the largest pocket size. Based on the charge distribution, the N-lobe contained mostly negative charges, whereas the C-lobe included positive charges due to a lysine residue. Intriguingly, the charge distribution pattern of CLK2 was the weakest compared with that of CLK1 and CLK3, indicating its preference to bind to the hydrophobic benzonaphthyridine ring of CX-4945 (Figure 5(b)). Therefore, based on the size and electrostatic charge distribution of the active site, the binding of CLK2 to CX-4945 could be stronger than the binding of the other two CLKs.

The low binding affinity for CLK3 has been previously explained by the Lerchner group [29]. As described in the previous section, the hydrophobic valine residue (Val324 and Val326 in CLK1 and CLK2, respectively) was substituted with alanine (Ala319) in CLK3. This substitution could result in the weaker hydrophobic interactions compared with the interactions of CLK1 and CLK2. Therefore, the binding affinities towards CLKs were in the following decreasing order: CLK2 > CLK1 > CLK3, which are consistent with previous findings [21, 29].

We also compared the binding mode of CX-4945 for CLK2 and CK2. The amino acid sequences of CK2 and CLK2 showed low similarity with 15.9% identity (Supp_Figure 3). Structural superposition of the CK2/CX-4945 (PDB code; 3PE1) and CLK2/CX-4945 complexes by Dali server showed that the overall structures were well overlaid with a Z-score of 28.3 and an r.m.s.d. of 2.6 Å aligned with 282 C*α* positions (Figure 5(d)). Therefore, the positions of CX-4945 were almost identical in the two overlaid structures. The active site of the CX-4945/CK2 complex revealed the involvement of four water molecules in mediating interactions between carboxylic acid as well as the benzonaphthyridine ring of CX-4945 and CK2 (Figure 5(e)) [30]. The amino acids Val53, Val66, Phe113, His115, and Val116 of the N-lobe and H160 and Ile174 of the C-lobe were stacked with the benzonaphthyridine ring of CX-4945 with hydrophobic interactions. Lys68 and the main chains of Val116 and Asp175 directly interacted with carboxylic acid and the naphthyridine ring of CX-4945 by hydrogen bonding. Therefore, Glu81, Asn118, His160, and Trp176 interacted with CX-4945 by water-mediated hydrogen bonding. Overall, the different active site environments of CLK2 and CK2 contributed to the different interactions and binding affinities.

## 4. Conclusion

In the present study, we determined the structures of CLK1, CLK2, and CLK3 in complex with their small molecule inhibitor CX-4945. Overall structure of CLKs was similar to each other, but a close look into the active sites revealed the notable difference in pocket sizes and electrostatic surface charge distributions (Figure 5). First, the active site of CLK1 was somewhat narrow and contained a negatively charged patch. Second, CLK3 had a protruded Lys248 residue in the entrance of the active site pocket, which might be unfavorable for the entry of CX-4945. In addition, Ala319, equivalent to Val324 (CLK1) and Val326 (CLK2), likely caused the weaker hydrophobic interactions with the benzonaphthyridine ring of CX-4945. Third, out of three CLKs, CLK2 has the weakest charge distribution, favoring its hydrophobic interactions with benzonaphthyridine ring. Together, these results support the relatively favorable binding of CX-4945 with CLK2 over CLK1 and CLK3, which is consistent with its stronger effect on CLK2 that were defined in the previous study.

## Figures and Tables

**Table 1:** Data collection and refinement statistics for CLKs in complex with CX-4945.

	CLK1/CX-4945	CLK2/CX-4945	CLK3/CX-4945
*Data collection*			
Space group	*P*2_1_	*P*4_3_2_1_2	*I*222
Cell dimensions			
a, b, c (Å)	56.5, 115.7, 90.6	75.6, 75.6, 161.8	61.8, 115.0, 158.3
*α*, *β*, *γ* (°)	90, 100.4, 90	90, 90, 90	90, 90, 90
Resolution range (Å)	50–2.7 (2.8–2.7)^a^	50–2.8 (2.9–2.8)	50–2.6 (2.69–2.6)
*R*_merge_ (%)^b^	15.9 (199.1)	18.9 (136.7)	15.4 (67.2)
*I */ *σI*	23.8 (2.4)	16.5 (2.0)	12.8 (2.3)
Unique reflection	31866	12177	17657
Completeness (%)	99.8 (99.7)	99.6 (99.5)	99.7 (100.0)
Redundancy	7.0 (7.1)	18.5 (18.6)	6.9 (5.8)
CC_1/2_^c^	0.988 (0.703)	0.962 (0.837)	0.987 (0.418)
Wilson B-factor	56.2	51.3	34.6
*Refinement*			
Resolution	31.9–2.7	40.5–2.8	24.4–2.6
No. of reflections	32028	12156	17642
*R*_work_^d^/*R*_free_^e^ (%)	20.0/27.4	19.6/26.1	22.3/28.4
No. of atoms	7711	2907	2780
protein	7614	2848	2689
ligand	75	25	25
water	22	34	66
B-factors			
protein	63.9	53.5	50.1
ligand	82.7	54.9	52.4
water	60.5	47.2	43.5
R.m.s. deviations			
bond lengths (Å)	0.009	0.008	0.010
bond angles (°)	1.29	1.03	1.38
PDB code	6KHD	6KHE	6KHF

^a^The numbers in parentheses are statistics from the highest resolution shell.

^b^ *R*_merge_ = Σ|*I*_obs_ − *I*_avg_|/*I*_obs_, where *I*_obs_ is the observed intensity of individual reflections and *I*_avg_ is averaged over symmetry equivalents.

^c^[22].

^d^ *R*_work_ = Σ||*F*_o_| − |*F*_c_||/Σ|*F*_o_|, where |*F*_o_| and |*F*_c_| are the observed and calculated structure factor amplitudes, respectively.

^e^ *R*_free_ was calculated using 10% of the data.

## Data Availability

The data used to support the findings of this study are available from the corresponding author upon request.

## Supplementary Materials

Supplementary Materials
*Supplementary Figure 1. Sequence alignment of the four isoforms of CLKs*. Amino acid sequences of the CLK1, CLK2, CLK3, and CLK4 proteins are aligned, and 100% identical residues are colored in red. Identical residues in CLK1 and CLK4 are colored in blue. The *α*-helix of CLK1 is colored in sky blue, and *β*-sheet is colored in purple. The common features of CLK are shown in solid line boxes (*β*-hairpin insertion: yellow; EHLAMMERILG motif: light green; MAPK-like insertion: gray).* Supplementary Figure 2. Structural comparison of CLKs with the inhibitors CX-4945 and KH-CB19*. (A) Superimposed structures of CLK1/CX-4945 (blue) and CLK1/KH-CB19 (PDB code, 2VAG; gray) structures. The CX-4945 and KH-CB19 molecules are colored in yellow and green, respectively. (B) Superimposed structures of CLK3/CX-4945 (magenta) and CLK3/KH-CB19 (PDB code, 2WUT; gray). The CX-4945 and KH-CB19 molecules are colored in yellow and green, respectively. Nitrogen, oxygen, and chlorine are colored in blue, red, and black, respectively.* Supplementary Figure 3. Structure-based sequence alignment of CLK2 and CK2*. Amino acid sequences from CLK2 and CK2 are aligned. Identical amino acids between CLK2 and CK2 are shown in red. The *α*-helices and *β*-sheets (secondary structures) of CLK2 are colored in yellow and dark yellow, respectively. The *α*-helices and *β*-sheets of CK2 are shown in light green and cyan, respectively.
