# Supplementary material for: Structural Basis for the Selective Inhibition of Cdc2-Like Kinases by CX-4945
Source: Biomed Res Int. 2019 Aug 18;2019:6125068. doi: 10.1155/2019/6125068 (PMC6720368; doi:10.1155/2019/6125068)
Supplement: Supplementary Materials — Supplementary Figure 1. Sequence alignment of the four isoforms of CLKs. Amino acid sequences of the CLK1, CLK2, CLK3, and CLK4 proteins are aligned, and 100% identical residues are colored in red. Identical residues in CLK1 and CLK4 are colored in blue. The α-helix of CLK1 is colored in sky blue, and β-sheet is colored in purple. The common features of CLK are shown in solid line boxes (β-hairpin insertion: yellow; EHLAMMERILG motif: light green; MAPK-like insertion: gray). Supplementary Figure 2. Structural comparison of CLKs with the inhibitors CX-4945 and KH-CB19. (A) Superimposed structures of CLK1/CX-4945 (blue) and CLK1/KH-CB19 (PDB code, 2VAG; gray) structures. The CX-4945 and KH-CB19 molecules are colored in yellow and green, respectively. (B) Superimposed structures of CLK3/CX-4945 (magenta) and CLK3/KH-CB19 (PDB code, 2WUT; gray). The CX-4945 and KH-CB19 molecules are colored in yellow and green, respectively. Nitrogen, oxygen, and chlorine are colored in blue, red, and black, respectively. Supplementary Figure 3. Structure-based sequence alignment of CLK2 and CK2. Amino acid sequences from CLK2 and CK2 are aligned. Identical amino acids between CLK2 and CK2 are shown in red. The α-helices and β-sheets (secondary structures) of CLK2 are colored in yellow and dark yellow, respectively. The α-helices and β-sheets of CK2 are shown in light green and cyan, respectively. [file 6125068.f1.pdf]

Supplementary Materials

Structural basis for the selective inhibition of Cdc2-like kinases by CX-4945

Joo Youn Lee[1,6], Ji-Sook Yun[1,6], Woo-Keun Kim[2], Hang-Suk Chun[2], Hyeonseok Jin[3], Sungchan Cho[4, 5*], and Jeong Ho Chang[1,3*]

[1]Department of Biology Education, Kyungpook National University, 80 Daehak-ro, Buk-gu, Daegu 41566, South Korea; [2]Biosystem Research Group, Korea Institute of Toxicology, 141 Gajeong-ro, Yuseong-gu, Daejeon 34114, South Korea; [3]Research Institute for Phylogenomics and Evolution, Kyungpook National University, 80 Daehak-ro, Buk-gu, Daegu 41566, South Korea; [4]Natural Medicine Research Center, Korea Research Institute of Bioscience and Biotechnology, 30 Yeongudanji-ro, Ochang-eup, Cheongju-si, Chungbuk 28116, South Korea; [5]Department of Biomolecular Science, Korea University of Science and Technology, 217 Gajeong-ro, Gajeong-dong, Yuseong-gu, Daejeon 34113, South Korea

[6]These authors contribute equally to this work

Running title: Crystal structures of CLK isoforms in complex with CX-4945

Keywords: Cdc2-like kinase, Casein kinase, CX-4945, Alternative splicing, X-ray crystallography

*Correspondence information

Sungchan Cho

Phone: +82-43-240-6105, E-mail: Sungchan@kribb.re.kr

Jeong Ho Chang

Phone: +82-53-950-5913, E-mail: jhcbio@knu.ac.kr

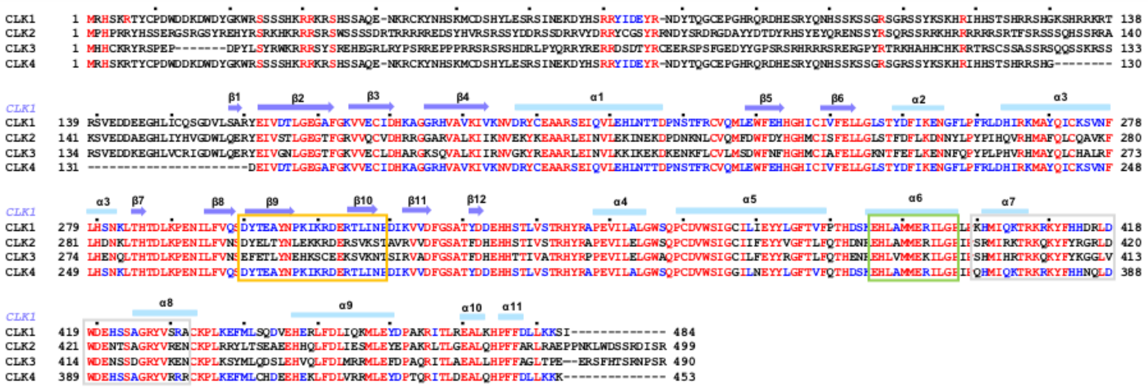

Supplementary Figure 1. Sequence alignment of the four isoforms of CLKs. Amino acid sequences of the CLK1, CLK2, CLK3, and CLK4 proteins are aligned, and 100% identical residues are colored in red. Identical residues in CLK1 and CLK4 are colored in blue. The α-helix of CLK1 is colored in sky blue, and β-sheet is colored in purple. The common features of CLK are shown in solid line boxes (β-hairpin insertion: yellow; EHLAMMERILG motif: light green; MAPK-like

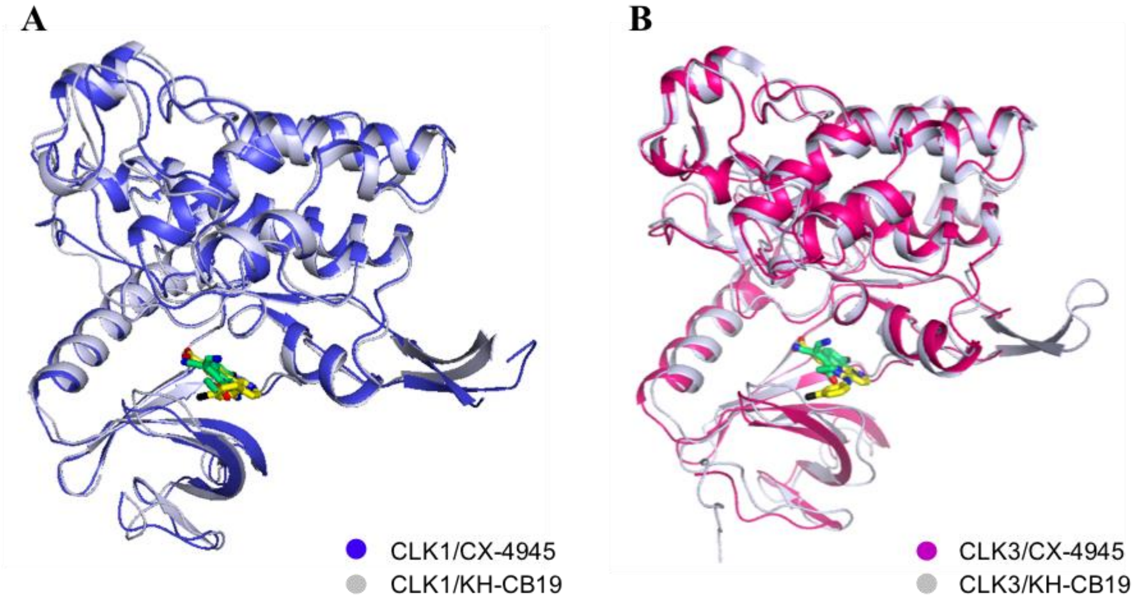

Supplementary Figure 2. Structural comparison of CLKs with the inhibitors CX-4945 and KH-CB19. (A) Superimposed structures of CLK1/CX-4945 (blue) and CLK1/KH-CB19 (PDB code, 2VAG; gray) structures. The CX-4945 and KH-CB19 molecules are colored in yellow and green, respectively. (B) Superimposed structures of CLK3/CX-4945 (magenta) and CLK3/KH-CB19 (PDB code, 2WUT; gray). The CX-4945 and KH-CB19 molecules are colored in yellow and green, respectively. Nitrogen, oxygen, and chlorine are colored in blue, red, and black, respectively.

```
CLK2
CLK2      1 MPHPRRYHSSERGSRGSYREHYRSRKHKRRRSRSWSSSSDRTRRRRREDSYHVRSRSSYD  60
CK2       1 ----------------------MSGPVPSRARVYTDVNTHRPREYWDYESHVVEWGNQD  37
CK2                                                        α1      β1

CLK2
CLK2     61 DRSSDRRVYDRRYCGSYRRNDYSRDRGDAYYDTDYRHSYEYQRENSSYRSQRSSRRKHRR 120
CK2      38 DYQLVRKLGRGKYSEVFEAINITNNEKVVVKILKPVKKKKIKREIKILENLRGGPNIITL  97
CK2            β2        β3        β4            α2

CLK2                                                β1    β2      β3
CLK2    121 RRRRSRTFSRSSSQHSSRRAKSVEDDAEGHLIYHVGDWLQERYEIVSTLGEGTFGRVVQC 180
CK2      98 ADIVKDPVSRTPALVFEHVNN-------------TDFKQLYQTLTDYDIRFYMYEILKA 143
CK2           β5      β6                             α3       α4

CLK2             β4             α1                         β5
CLK2    181 VDHRRGGARVALKIIKNVEKYKEAARLEINVLEKINEKDPDNKNLCVQMFDWFDYHGHMC 240
CK2     144 LDYCHSMGIMHRDVKPHNVMIDHEHRKLRLIDWGLAEFYHPGQEYNVRVASRYFKGPELL 203
CK2                β7          β8    β9     β10                 α5

CLK2          β6     α2             α3             β7         β8
CLK2    241 ISFELLGLSTFDFLKDNNYLPYPIHQVRHMAFQLCQAVKFLHDNKLTHTDLKPENILFVN 300
CK2     204 VDYQMYDYSLDMWSLG----------CMLASMIFRKEPFFHGHDNYDQLVRIAKVLGTE 252
CK2                         α6                    α7

CLK2          β9         β10   β11   β12                α4
CLK2    301 SDYELTYNLEKKRDERSVKSTAVRVVDFGSATFDHEHHSTIVSTRHYRAPEVILELGWSQ 360
CK2     253 DLYDYIDKYNIELDPR-------FNDILGRHSRKRWERFVHSENQHLVSPEALDFLDK-- 303
CK2           α8                α9                         α10

CLK2               α5              α6         α7       β13 β14
CLK2    361 PCDVWSIGCIIFEYYVGFTLFQTHDNREHLAMMERILGPIPSRMIRKTRKQKYFYRGRLD 420
CK2     304 -----------------LLRYDHQSR--------------LTAREAMEHPYFYTVVKD 330
CK2                                                   α11     α12

CLK2                α8              α9                   α10
CLK2    421 WDENTSAGRYVRENCKPLRRYLTSEAEEHHQLFDLIESMLEYEPAKRLTLGEALQHPFFA 480
CK2     331 QARMGSS-----SMPGGSTPVSSANMMSGISSVPTPSPLGPLAGSPVIAAANPLGMPVPA 385
CK2

CLK2          α11
CLK2    481 RLRAEPPNKLWDSSRDISR 499
CK2     386 AAGAQQ------------- 391
CK2
```

Supplementary Figure 3. Structure-based sequence alignment of CLK2 and CK2. Amino acid sequences from CLK2 and CK2 are aligned. Identical amino acids between CLK2 and CK2 are shown in red. The α-helices and β-sheets (secondary structures) of CLK2 are colored in yellow and dark yellow, respectively. The α-helices and β-sheets of CK2 are shown in light green and cyan, respectively.
